# Initial Implementation of a Comparative Data Analysis Ontology

**DOI:** 10.4137/ebo.s2320

**Published:** 2009-07-03

**Authors:** Francisco Prosdocimi, Brandon Chisham, Enrico Pontelli, Julie D. Thompson, Arlin Stoltzfus

**Affiliations:** 1 Department of Structural Biology and Genomics, Institut de Génétique et de Biologie Moléculaire et Cellulaire (IGBMC), F-67400 Illkirch, France; Institut National de la Santé et de la Recherche Médicale (INSERM), U964, F-67400 Illkirch, France; the Centre National de la Recherche Scientifique (CNRS), UMR7104, F-67400 Illkirch, France; Université de Strasbourg, F-67000 Strasbourg, France; 2 Department of Computer Science, New Mexico State University, P.O. Box 30001, MSC CS, Las Cruces, NM 88003, USA; 3 Center for Advanced Research in Biotechnology, University of Maryland Biotechnology Institute, 9600 Gudelsky Drive, Rockville, Maryland 20850, USA; Biochemical Science Division, National Institute of Standards and Technology, Mail Stop 8310, Gaithersburg, Maryland, 20899-8310, USA. Email:arlin.stoltzfus@nist.gov

**Keywords:** evolution, phylogeny, ontology, character data, comparative method

## Abstract

Comparative analysis is used throughout biology. When entities under comparison (e.g. proteins, genomes, species) are related by descent, evolutionary theory provides a framework that, in principle, allows N-ary comparisons of entities, while controlling for non-independence due to relatedness. Powerful software tools exist for specialized applications of this approach, yet it remains under-utilized in the absence of a unifying informatics infrastructure. A key step in developing such an infrastructure is the definition of a *formal ontology*. The analysis of use cases and existing formalisms suggests that a significant component of evolutionary analysis involves a core problem of inferring a character history, relying on key concepts: “Operational Taxonomic Units” (OTUs), representing the entities to be compared; “character-state data” representing the observations compared among OTUs; “phylogenetic tree”, representing the historical path of evolution among the entities; and “transitions”, the inferred evolutionary changes in states of characters that account for observations. Using the Web Ontology Language (OWL), we have defined these and other fundamental concepts in a *Comparative Data Analysis Ontology (CDAO).* CDAO has been evaluated for its ability to represent token data sets and to support simple forms of reasoning. With further development, CDAO will provide a basis for tools (for semantic transformation, data retrieval, validation, integration, etc.) that make it easier for software developers and biomedical researchers to apply evolutionary methods of inference to diverse types of data, so as to integrate this powerful framework for reasoning into their research.

## Introduction

High-throughput techniques have led to a rapid increase in the availability of various types of biological data. These data do not speak for themselves, but may serve as inputs for methods that generate clues or inferences. One of the main methods used for generating such inferences is the *comparative* approach. For instance, when a new genome sequence is determined, an enormous amount of useful information is revealed by comparing it with other genomes and interpreting *patterns of similarity and difference*, with applications in identifying regulatory sites,^1^ predicting protein structures,^2^ and interpreting SNP variation.^3^

Because similarities and differences among biological entities emerge by a process of descent with modification, evolutionary theory provides a mechanistic framework for interpreting biological comparisons. An evolutionary approach to comparisons emerged over several decades from the efforts of taxonomists to replace personal judgment with rigorous principles.^4^,^5^ This approach can be distilled to three principles. First, an evolutionary analysis begins by identifying relationships, not just of similarity, but of similarity due to descent-with-modification from common ancestors, i.e. evolutionary *homology*. Second, as astute bioinformaticians often emphasize with frustration, common statistical methods that would treat evolved entities as independent samples are inappropriate: evolved entities are *not* independent, but have a tree-like structure of relationships, making phylogenetic trees essential for any rigorous analysis.^6^,^7^ Evolutionary methods control for relatedness (non-independence) because they “exploit phylogenies to reveal independent events of evolution”.^8^ Third, the events of change (along the phylogeny) that are invoked to account for observed biological differences are not ordinary transformations of biological substances (i.e. not like the development of an embryo, or the formation of a scar), but *evolutionary transitions* that follow the rules of evolutionary genetics, with any accompanying biases due to the dynamics of mutation, genetic transmission, and reproductive sorting (selection and drift).

The evolutionary approach is not the only possible approach to comparisons. A common alternative to analyze comparative data is to apply generic methods of classification or machine-learning, such as neural networks and support vector machines,^9^ that rely on a simple principle of similarity (e.g. protein X is a dehydrogenase because its sequence looks like that of other dehydrogenases) or on “guilt by association” (e.g. protein X is involved in mercury resistance because the gene encoding X is linked chromosomally to gene Y involved in mercury resistance). Relative to such heuristic approaches, the promise of evolutionary methods is that, because they incorporate a model of the actual generative process underlying the data, they will be more accurate and flexible, and particularly useful for cases in which the outcome of evolution departs significantly from the expectations of a purely functional approach, e.g. whenever mutation biases are important.^10^ The full application of a comparative method based on evolutionary theory makes it possible to refine relatively vague and difficult questions about how to interpret similarities and differences into more well-posed questions about rates and processes of change along the branches of a phylogenetic tree, e.g. providing a basis to assign probabilities to unknown states, such as the activity or co-factor-specificity of an enzyme.^11^

In spite of their clear advantages, evolutionary analyses remain under-utilized. This may reflect a need to educate researchers on the generality of evolutionary methods. However, it also suggests a need to reduce technical barriers. The traditional computational approach to evolutionary analysis is for an expert user to manually shepherd a single set of data through a series of steps relying on domain-specific software, often with idiosyncratic interfaces, and requiring a variety of user interventions to extract intermediate results, trap errors, and customize operations. This expert-supervised approach is time-consuming, error-prone, difficult to document (and, thus, to validate or to reproduce), and therefore represents a barrier to large-scale, integrative, or multidisciplinary analyses.

The existence of substantial technical barriers is apparent from the development of methods for assigning “functions” to proteins encoded by newly determined genome sequences. Soon after this problem emerged as a major computational challenge,^12^ Eisen presented compelling arguments (by reasoning from case studies) that accurate assignments would require a phylogenetic framework, not merely identification of a “best hit” via BLAST searches.^13^ Nevertheless, genome annotation projects continued to develop and apply approaches based on similarity and guilt-by-association. Years went by before approximations of Eisen’s rule-based “phylogenomics” framework were automated;^14^,^15^ and only recently Englehardt, et al developed an explicit and generalized probabilistic model^11^ to replace rule-based reasoning. Meanwhile, new problems amenable to the evolutionary approach continue to emerge, e.g. the inference of interactions between sites within a protein,^16^ or between different proteins;^17^ or the inference of changes in gene expression.^17^,^18^

An integrated solution to lower the barrier for applying an evolutionary approach might make use of a combination of technologies, including applications software, web services,^19^ workflow systems,^20^ data standards, and ontologies.^21^,^22^ Powerful applications software already exists already for many steps in evolutionary analysis. Access to these tools can be greatly enhanced through the use of web services and other software services, as in the myGrid^23^ and BioMoby^24^ projects. However, to assemble these services into fully automatic workflows requires a way to standardize knowledge, thus facilitating data re-use and data interoperability.

In recent years, the utility of ontologies for standardizing knowledge has been widely demonstrated,^25^–^28^ but the role of ontologies remains widely misunderstood. A common misconception (which emerged in the review of this paper) is that an ontology is a special kind of file format, or that a well defined data format obviates the need for an ontology. Actually ontologies and file formats address different problems. Data formats, which are designed to provide a concrete representation of data for purposes of storage or exchange, represent a form of *syntax* for “writing down” data. In contrast, an ontology focuses on *semantics*, that is, the meaning of the data; an ontology expressed in a given language is not necessarily tied to a specific file format (e.g. OWL statements are commonly represented in RDF/XML, but there is also an OWL functional syntax). An ontology contains not only the vocabulary (terms and labels), but also the definition of the concepts and their relationships for a given domain. To illustrate this important distinction, let us imagine a simple FASTA file:

>AMYLASEE

TGCATNGY

A problem with this representation of data is that a computer does not have access to the semantics. By convention, a FASTA file has an identifier line (sometimes called the “definition line”) starting with “>” and ending with a newline, followed by a sequence. Thus, a human expert would understand that the string “TGCATNGY” must be some kind of sequence, but could not tell if it is a DNA sequence (Thymine, Guanosine, …) or a protein sequence (Threonine, Glycine …), since the symbols could come from either the commonly used alphabet for DNA residues, or that for amino acid residues. Likewise, a human expert would understand that the string “AMYLASEE” is an identifier, but not what it means in relation to the sequence: it might be “Amylase E”, representing the name of a gene or protein, or it might refer to “Amy Lasee”, the name of a donor or an experimenter—or it might mean something else.

An XML version of the above FASTA example might look like this, noting that a FASTA archive may have multiple sequence records:


    <xml>

          <fasta_archive>
                <fasta_record>
                      <identifier>**AMYLASEE**</identifier>
                      <sequence>**TGCATNGY**</sequence>
                </fasta_record>
          </fasta_archive>
    </xml>


Rendering the data in XML format, with a schema to validate against, makes the value of the strings “AMYLASEE” and “TGCATNGY” much more interpretable, because they can be accessed and validated by readily available tools on any computer platform. However, this does not solve any of the problems of semantics noted above. We might imagine that adding extra tags would solve the problem:


    <fasta_record>
              <identifier>
                   <protein_name>**AMYLASEE**
                   </protein_name>
              </identifier>
              <sequence>
                   <protein_sequence>**TGCATNGY**
                   </protein_sequence>
              </sequence>
    </fasta_record>


But this does not formalize the semantics or make them accessible to a computer—unlike a human expert, the computer cannot supply the meanings hidden in the tag names, and only sees arbitrary strings like this:


    <string1>
            <string2>
                    <string3>**AMYLASEE**</string3>
            </string2>
            <string4>
                    <string5>**TGCATNGY**</string5>
            </string4>
    </string1>


How can we make it clear that “TGCATNGY” represents the sequence of amino acid residues in a protein? How can we explain the relationship between the name and the sequence? Ontologies are designed specifically to solve this kind of problem by encoding or formalizing knowledge in a computable form that can be referenced when data are described. If “TGCATNGY” is a protein sequence, we might express this by referring to SO:0000104, the “polypeptide region” concept in the Sequence Ontology;^29^ or we might refer to the fourth residue not with the character “A”, but with a reference to CHEBI:32433, which is the CHEBI (Chemical Entities of Biological Interest;)^30^ term for the L-Alanyl moiety in a polypeptide chain.

In order to provide a formalization of knowledge that could serve as a basis for improving interoperability in comparative analysis, we initiated the design and development of a suitable ontology. From the analysis of use cases (i.e. specific tasks representing the widely used methods in evolutionary analysis) and related artefacts (e.g. file formats, database schemas, software interfaces, and so on), the inference of *character histories* emerged as the core problem in evolutionary comparative analysis, relying on the concepts of *phylogenetic tree*, *Operational Taxonomic Unit*, *character-state data,* and *transition* (i.e. an evolutionary change in the state of a character). These important concepts were formalized using the standard Web Ontology Language (OWL)^31^ to build a prototype version of a *Comparative Data Analysis Ontology (CDAO)*. An initial evaluation of the prototype has also been performed, encoding token data sets as CDAO instances and implementing simple query and reasoning tasks. The development of CDAO will continue in the context of supporting specific research objectives and we anticipate that, in the near future, CDAO will help to improve data interoperability in evolutionary methods and to lower the technology barrier for applying an evolutionary approach to comparative analyses.

## Methods

### Development strategy

**Figure 1:**
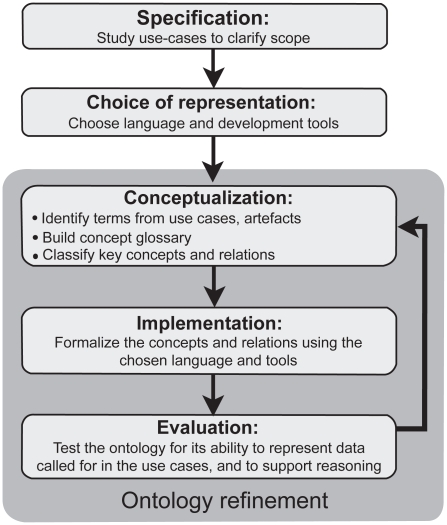
ontology development strategy. The strategy for development of CDAO was modified from that suggested by Stevens et al.^32^ We began by studying use cases. After deciding on a representation system, we conceptualized domain knowledge by identifying, defining, and classifying terms for key concepts and relations. These concepts and relations were formalized, and then subjected to evaluation as described in the text.

The strategy adopted to develop CDAO (Fig. 1), roughly followed the ontology building life-cycle suggested by Stevens et al.^32^ We began (*Specification* step) by considering tasks and use cases, ranging from every-day chores, e.g. sequence alignment, to challenging projects, e.g. comparing developmental gene expression patterns across species.^33^ At the same time we gathered a list of related artefacts—file formats, database schemas, software interfaces, and so on—that have been proposed or are in use in the evolutionary analysis domain (some are listed in Table 1).

For the subsequent *Conceptualization* step, we identified key concepts and the relationships between them by studying these use cases and related artefacts. Distinctive terms were identified manually and used to populate a concept glossary. Definitions for relevant glossary terms were developed by studying usage in articles and books (e.g.^4^,^5^,^34^,^35^) by consulting domain experts (in the Evolutionary Informatics working group sponsored by the National Evolutionary Synthesis Center, NESCent), and by studying the use of terms in phylogenetic software interfaces.

Of the several languages available to formalize concepts and relations, we chose the Web Ontology Language (OWL)^31^ and more specifically OWL version 1.1.^66^ OWL is a language for the description of ontologies (in terms of concepts, properties, and annotations), which builds on the solid theoretical foundations of Description Logics,^80^ a class of logics for describing and reasoning about concept descriptions. OWL, apart from being an accepted standard in the world of ontologies, has the added advantage of providing access to sophisticated querying and reasoning engines. In particular, OWL 1.1 provides features such as inverse properties, transitive properties, and property chaining (discussed later) that are helpful for encoding the type of knowledge encountered in this work. We implemented classes and relations in OWL using the free, open-source editor Protégé 4,^36^ chosen for its support of OWL 1.1 and its plugins for the Pellet^37^ reasoner (which can be used to infer relationships, making use of OWL 1.1 features) and for GraphViz (to visualize the hierarchy of classes).

### Evaluation

CDAO has been evaluated for its ability to represent data and to support simple forms of querying and reasoning. The data representation capability has been tested by developing software to translate comparative data encoded in NEXUS format^38^ to instances of CDAO concepts and relations. NEXUS is a well-established standard format, supported by libraries in C++^39^ and Perl,^40^ and by various applications (e.g. PAUP*^41^ and MrBayes^42^). The output of the translation process is a description of the data in the input files as instances of the concepts and properties in CDAO; the instances are presented as triples (subject-property-object), encoded using the standard Resource Description Framework (RDF) triple representation.^43^

One translation tool, written in C++ using the NEXUS Class Library (NCL)^39^ extracts information from NCL classes to populate an internal object representation that more closely matches CDAO classes. This internal representation is traversed to generate instances of CDAO classes and properties, expressed as RDF/XML statements. A second translation tool was developed using the Perl Bio:: NEXUS library^40^ to process input NEXUS files and extract information, expressed in a flat database format. This flat database is then processed by a Prolog^44^ program to generate the RDF/XML statements according to CDAO. In both cases, the validity of the generated data instances was checked manually by verifying the output of examples that (collectively) encompass the set of desired translation features.

For the representation tests and the reasoning tests (described below), we employed 8 data sets each including a single matrix of character data and one or more phylogenetic trees. The data matrices included smaller (10 sequences) and larger (47 sequences) versions of both the protein sequence alignment and the coding sequence alignment for members of the ATP-synthetase C family (Pfam PF00137), protein and coding-sequence alignments for 39 members of the cytochrome C family (Pfam PF00034), sensitivity (on a continuous scale) to 20 chemical inhibitors for a set of 115 human kinases (kindly provided by Dr. James R. Brown), and comparative data on worm development and anatomy (51 characters, 55 species).^45^

While traditional databases and XML formats only allow posing queries that extract data (e.g. using SQL), ontologies impose a semantic layer that enables queries that involve *reasoning*. Protégé allowed the development of simple reasoning tasks, which were carried out interactively using the *DL Query* window. These reasoning tasks are performed using the plug-in reasoners FaCT++^46^ and Pellet.^37^ We also developed a small automated test suite, based on scripts that call the external reasoning engine, *Pellet*,^37^ and another set of scripts using the *Racer* engine.^47^ This more systematic reasoning test includes a set of queries aimed at extracting information from the data instances. In order to verify that artificial information was not introduced by the representation, some queries have been designed to produce no results. The queries are expressed in the SPARQL syntax,^48^ a query notation that is designed to operate on RDF data, and that is supported by the Pellet reasoner. The scripts execute queries described in the Evaluation section of Results. Complete test results are available at http://www.cs.nmsu.edu/~bchisham/ontology/test_results/.

### Availability of products

The CDAO ontology is available (under the terms of the GNU GPL) in OWL XML/RDF format from the evolutionary ontology web site at www.evolutionaryontology.org (the CDAO code repository is maintained at SourceForge; http://sf.net/projects/cdao). The web site includes documentation on CDAO’s classes and relations, as well as a translator from NeXML to CDAO RDF/XML, based on XSL Transformation (XSLT; Clark 1999); NeXML is a recently proposed XML standard for the encoding of phylogenetic data.^49^

## Results

### Domain specification

#### Use cases

We began with a list of use cases (http://www.nescent.org/wg_phyloinformatics/UseCases) developed for a previous project, the NESCent Phyloinformatics hackathon,^50^ and refined by the NESCent Evolutionary Informatics working group. The description of each use case includes background, references, major steps, sample data, and key challenges. The use cases are associated with three kinds of research goals:

1. Obtaining an objective classification for compared entities, as in classical or contemporary systematics;
2. Understanding the history of evolution, including both a chronicle of events and an explanation of motivating causes;
3. Making biological inferences about the present.

These objectives are not exclusive; for example, attempts to understand the evolution of a character often have direct implications for understanding the present-day biology of the same character. Felsenstein^34^ (p. 145) argues that, in actual practice, most “systematists” are not concerned primarily with classification (goal 1), but with the biology and evolution of characters (goals 2 and 3).

The use cases are not atomic, but typically consist of a sequence of operations, many of which recur in other use cases. For instance, analyses of molecular sequence data typically begin with a common set of steps (e.g. as in^51^):

- Identify related sequences (“homologs”) using database queries;
- Derive a multiple alignment of the homologous sequences;
- Prune or edit the resulting alignment;
- Infer a phylogenetic tree.

Similar operations may be applied to different goals and different types of data. For instance, morphological attributes may be assigned discrete values and analyzed by the same types of rule-based (e.g. parsimony) or probabilistic (e.g. maximum-likelihood) methods that are applied to discrete molecular characters, such as nucleotide residues in a sequence alignment.

#### Relevant artefacts

The analysis of existing artefacts serves several purposes: identifying preferred terms associated with key concepts, studying previous strategies for representation, and identifying overlaps and redundancies. We considered a range of artefacts (some of them listed in Table 1), including previously defined ontologies, database schemas, file formats, and software interfaces.

The artefacts used most in evolutionary analysis focus on a generic *syntax* for characters, without significant biological depth. For instance, widely used data formats for sequence alignments such as PHYLIP,^52^ MEGA,^53^ or ClustalW,^54^ focus on a narrow syntax for nucleotide or protein sequences, with no other biological features and with no metadata other than sequence names. NEXUS files may be used to encode phylogenies as well as comparative molecular or morphological data; the representation of comparative data consists of a matrix of symbols that may be assigned labels but that otherwise lacks semantics. The format description for NEXUS^38^ provides for greater biological depth for some types of molecular characters (e.g. to represent a genetic code and to specify the reading frame of a protein-coding sequence), but such advanced features of NEXUS are rarely used. In contrast, the data model underlying NCBI services^55^ is extraordinarily rich with regard to molecular sequences and associated information, including alignments, sequence “features”, and so on. Many of these elements are present in CHADO,^56^ and can be represented in terms of the Sequence Ontology, SO.^29^ The most relevant ontology encountered is the Multiple Alignment Ontology, MAO.^57^

Phylogenetic trees are not the main focus of any of these artefacts (Table 1). The input and output format used by domain scientists for phylogenetic trees is nearly always a text string of nested parenthetical statements with a simple syntax referred to as the “Newick” or “New Hampshire” format, e.g. *(cat, (rat, mouse))*, as explained on p. 590 of Felsenstein.^34^ This syntax is natural for the representation of directed (rooted) trees, with the outermost parentheses representing the deepest node (“root” of the tree). Nevertheless, the same syntax is also commonly (and confusingly) used to represent unrooted trees, sometimes by pre-pending the token “[&U]” to indicate *unrooted*.^38^ Proposed extensions or replacements, such as NeXML,^49^ NHX,^58^ and phyloXML,^59^ provide additional mechanisms to add arbitrary (but unregulated) annotations to nodes and branches.

None of these artefacts (Table 1) serves the role of an ontology in providing computable access to semantics; and available ontologies^60^ do not cover the domain of comparative analysis.

### Conceptualization

From the preliminary analysis of use cases and artefacts, we moved to a more detailed and intensive effort to identify and define domain-specific concepts.

#### Concept glossary

First, a *concept glossary* was developed, as a concerted effort of the NESCent Evolutionary Informatics working group (evoinfo.nescent.org/ConceptGlossary). The current glossary is limited to terms that denote general concepts (e.g. *phylogeny inference method*), not specific instances (e.g. *MrBayes*), and includes some promiscuous terms that have a domain-specific meaning (e.g. root, transition). Ambiguities and overlaps are ameliorated in some cases by qualifying domain-specific terms. For instance, in the context of evolutionary analysis, the concept *tree* typically refers to a phylogenetic tree, while *taxonomy* invariably refers to a classification of organisms— into sub-species, species, genera, families, and so on. Thus, the glossary has domain-specific entries for “phylogenetic tree” and “organismal taxonomy”. The definitions include some information on subsumption and synonymy. The current version of the glossary contains 110 defined terms and 28 undefined terms, and includes hyperlinked cross-references in the term descriptions.

#### Identification of a core problem

The analysis of use cases and artefacts suggested that the domain of evolutionary analysis revolves around the core problem of inferring a phylogenetic character history. A “*character*” is an attribute shared among the entities to be compared; its observed instances or values are called the “*states*” of the character. A character history accounts for the observed distribution of values of a character by invoking discrete value-shifts. In more precise domain-specific language, the core problem is to derive a phylogenetic reconstruction that accounts for the observed distribution of states of a character among a collection of entities, commonly referred to as Operational Taxonomic Units (OTUs), by invoking evolutionary transitions (transformations) along the branches of a phylogenetic tree.

The operations identified in the use cases can be understood as precursors, component parts, elaborations, or extensions of this core problem of character analysis. For example, sequence alignment is a precursor to character analysis: it is required to assign relationships of homology such that a given residue instance in a given sequence instance is aligned with the residue instances at corresponding (homologous) positions in the other sequence instances. For another example, the “reconcile tree” problem is an elaboration of the concept of inferring a character history, in which the character of interest (a gene *in toto*) is subject to duplication.

The core problem of inferring a character history pertains closely to the three main aims of evolutionary analysis: phylogenetic classification, evolutionary reconstruction, and biological inference. To reconstruct ancestral states, one needs a tree; such a tree is found typically by searching for the tree that, according to reconstructed character histories, renders the present data most likely. Thus, character analysis underlies phylogeny inference, which underlies classification. The problem of reconstructing an ancestral state and the problem of biological or “functional” inference (e.g. protein “function” assignment)^11^ are not simply related problems, in the context of statistical inference they *represent the same mathematical problem* of inferring an unknown state (whether a present-day state or an ancestral state) given a tree, a model of transitions, and some observed data.

#### Key concepts

The key concepts of phylogenetic character analysis focus on *(i)* the matrix of character-state data for a set of OTUs; *(ii)* phylogenetic trees and networks; and *(iii)* rules or models for evolutionary changes (transitions, transformations).

##### (i) Character-state data matrix

**Figure 2:**
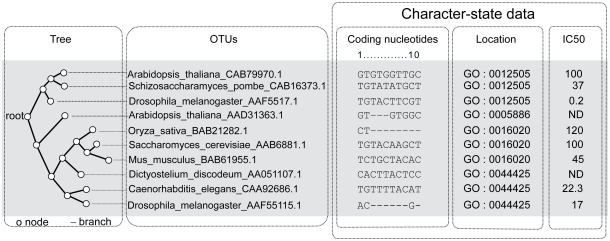
Illustration of some key concepts in evolutionary analysis. These data on a hypothetical family of proteins may be used to illustrate various concepts that are familiar in the domain of comparative evolutionary analysis. Phylogenetic trees have tips that typically represent currently existing biological entities (here proteins) that are referred to as OTUs, and that are associated with character-state data. The tips of the tree are linked to their ancestors (internal nodes) by branches or edges. Aligned sites in a protein-coding sequence are a type of character with a coordinate system (1 … 10) and with discrete states comprising nucleotides (A, T, C, G) or an alignment gap (−). Individual characters can be combined to form a compound character, e.g. 3 consecutive base-pairs combined to represent a single codon. The cellular location represented by a Gene Ontology (GO) term is also a discrete character that can be analyzed using the comparative evolutionary approach. An example of a continuous character would be the response of the protein to a chemical inhibitor (here shown as an IC50 value in micromolar). ND indicates that the state of a character is unknown for a given OTU.

Artefacts such as the NEXUS file format standard^38^ immediately suggest an Entity-Attribute-Value model that may be called the “character-state data model”,^40^ as shown in Figure 2. In this model, the entities to be compared are characterized by a table of data called a *“character data matrix”* (also known as *“character-state matrix”* or *“character-state data matrix”,* e.g. as in^38^). Each row of the matrix represents observations related to the same *OTU*. In classical systematics, OTUs typically are *species*, but they may be higher- or lower-level units such as genera or sub-species. In contemporary molecular studies, typically OTUs are genes or proteins, but may be other units, such as chromosomes or organelles. For each column, called a *“character”*, each OTU has a *“character-state”* (or, simply, a *“state”*).

The “character-state” data model is generic and can be applied to virtually any class of characters and OTUs, e.g. in a protein sequence alignment, the OTU represents a protein, the character represents a column in the alignment and the character state is either a residue or a gap. The states of high-level biological characters (morphology, development, anatomy, behavior) typically are encoded as discrete states, often with informative natural-language labels but no explicit semantics.^45^,^61^ Missing data and absent features, common in biological data, may be treated as an extra state. Thus, a feature that is found only in some OTUs, such as an intron at a particular site, can be rendered as a binary character with “presence” and “absence” states. In the example shown in Figure 2, the OTUs are proteins and each OTU is described by a number of different characters, including the coding nucleotide sequence, the cellular location and the response of the protein to a chemical inhibitor.

##### (ii) Phylogenetic trees and networks

In evolutionary biology, phylogenetic *trees* and *networks* are used to represent paths of descent-with-modification. The domain-specific meanings assigned to these terms are not the same as those used by mathematicians and computer scientists. In graph-theoretical terms, the canonical phylogenetic tree (see example in Fig. 2) is a connected, singly linked graph, in which the direction of the links goes from a single source node (the root) to multiple sink nodes (the terminals), in which each node has at most one parent (i.e. branches never fuse), and in which each node has zero or two children, (i.e. each branching is a bifurcation).

These restrictions reflect specific assumptions about the generating process, i.e. evolution. Since evolution moves forward in time, the branches (*edges*) on a tree are *directed*. The terminal nodes typically are anchored in the present time, because they represent observations or measurements made on currently existing organisms. The internal nodes represent common ancestors, with the deepest node as the “root” node of the tree. The restriction that each node has at most one immediate ancestor reflects the assumption that evolutionary lineages, once separate, do not fuse; such an assumption would follow from the “biological species concept” based on reproductive isolation.^62^ Branching is seen as a binary process of splitting by speciation or, in the case of molecular sequences, by gene duplication (the gene-wise equivalent of speciation). An instantaneous 3-way split is assumed to be impossible or vanishingly rare.

These typical or canonical restrictions sometimes are abandoned, either to allow for uncertainty in inferences, or because the biological assumptions do not hold. When a fully resolved bifurcating tree cannot be determined with sufficient reliability, so-called “*polytomies*” (nodes with more than two children) may be allowed. The availability of fossil evidence allows for terminal nodes that are not anchored in the present.^61^ Even with terminal nodes anchored in the present, it may be impossible to infer the direction of each internal branch, in which case the tree may be referred to as an *“unrooted tree,”* or as a *“network”.* Even the restriction of single parentage may be abandoned, for strictly biological reasons, in the case of lateral transfer (a partial mixing of lineages due to the transfer of one or a few genes),^63^ or in the case of reticulate evolution (a genome-level mixing of lineages due to interbreeding between previously separate species).^64^

##### (iii) Transitions, rules and models

Evolutionary change along the tree is understood to be a process of transition from one state to another, so that the interpretation of similarities and differences among evolved things becomes an issue of evolutionary dynamics (the model of change) along paths of descent (the tree). The practitioner’s understanding of evolutionary change is embodied in what may be called, at least in its more formal guises, a “transition model”. This model of transitions may range from informal justifications for assumptions about a few key characters of interest, as in Cavalier-Smith’s “transition analysis”,^65^ to a more formal set of constraints on allowable changes, to a rule-based variable weighting system (as in weighted parsimony), to a full probabilistic model. Ideally, the transition model applied to a set of characters reflects character-specific factors influencing evolutionary change. Especially for higher-level biological data, the expert’s understanding of character evolution may be crucial.^4^ However, often a generic model is used that makes minimal assumptions, e.g. allowing all possible changes to occur at the same rate.

### Implementation

The key concepts that emerged in the conceptual analysis have been formalized in OWL 1.1^66^ and implemented using the knowledge editor Protégé 4.

During the implementation of an ontology, the knowledge extracted from the conceptualization phase has to be encoded in terms of *concepts* (or *classes*), representing the classes of entities appearing in the domain, and *relations* (or *properties*), representing typed dependencies between concepts. Intuitively, an ontology describes each entity in a domain in terms of its “type” (i.e. the class it belongs to) and in terms of the characteristics of such an entity (i.e. the properties it has). Thus, each instance of a domain is essentially described by a collection of *subject*-*property-object* triples.

We will employ the notation *Property: Domain* → *Range*, to state that the given *Property* associates entities from the class *Domain* to entities from the class *Range*. We also use the notation Class_1_: Class_2_ to denote that Class_1_ is a subclass of Class_2_ (i.e. each entity of type Class_1_ is also of type Class_2_). The description of properties in OWL 1.1 may include not only the domain and range, but also additional characteristics that relate to *inverse properties*, *transitivity*, and *property chaining*. The inverse of a property *p* is another property *q* such that whenever the triple *subject-p-object* is present, then *object-q-subject* is implied, e.g. imagine that p is the relation “is upstream of ” and q is the relation “is downstream of”. If a property p is transitive, then whenever we have the triples *subject-p-object_1_* and *object_1_-pobject* *_2_*, then *subject-p-object_2_* is implied, e.g. this applies to ordering properties such as “is greater than”, or “precedes”. Given two properties *p_1_* and *p_2_*, property-chaining allow us to implicitly define a new property *p_3_*, such that the triple *subject-p_3_-object_1_* is implied whenever there exists another entity *object_2_* such that *subject-p_1_-object_2_* and *object_2_-p_2_-object_1_*, e.g. the father of my parent is my grandfather, so we can define “is grandfather of ” as the chain of two properties, “is father of ” and “is parent of ”.

#### Representation of core concepts and relations in CDAO

**Figure 3:**
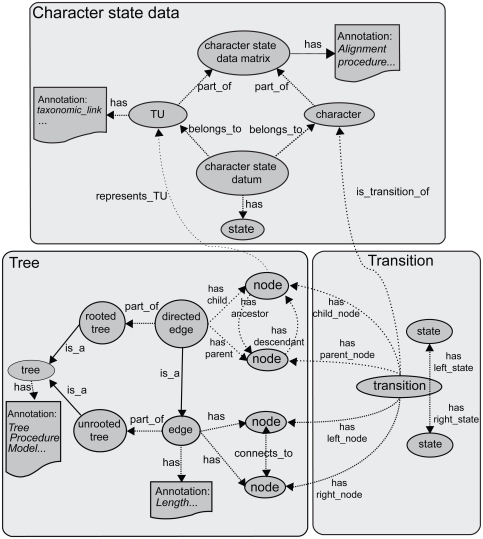
Some key concepts and relations formalized in CDAO. Domain-specific terms in CDAO represent either classes, shown by ovals and boxes, or properties (also called “relations”), shown by lines with arrows. The subsumption property “is_a” relates a class to its superclass (solid lines). other properties are defined in CDAO and discussed in the text (dashed lines).

The key concepts for the core problem of evolutionary character analysis include character-state data, OTUs, phylogenies, and transitions. Except for “transitions”, which for the present are represented as annotations of tree branches, these concepts have been implemented as separate class hierarchies in CDAO (Fig. 3), with a minimal number of relationships linking important concepts in each hierarchy. Each of these hierarchies is described in the rest of this section.

##### (i) Character-state data matrix

The representation of character-state data in CDAO is fine-grained. A ***CharacterStateDataMatrix*** has (via the ***has_Character*** property) ***Character***s and (via the ***has_TU*** property) ***TU***s, i.e.

*has_Character: CharacterStateDataMatrix* → *Character*

*has_TU: CharacterStateDataMatrix* → *TU*

The item representing a given ***Character*** in a given ***TU***, i.e. the item representing a cell in a character data matrix, is a ***CharacterStateDatum***: it ***belongs_to*** its ***TU***, ***belongs_to*** its ***Character***, and has a state-value, assigned by the ***has_State*** property, from an appropriate ***CharacterStateDomain***. For example, in Figure 2, the ***CharacterStateDatum*** that ***belongs_to TU*** A_thaliana_AAD31363.1 and ***belongs_to Character*** location ***has_State*** GO:0005886.

**TU** is a concept used to represent *Taxonomic Units*, i.e. individual units of analysis associated with rows in a character-state data matrix. TU subsumes the traditional concepts of Operational Taxonomic Unit (OTU)—typically associated to terminal nodes of a phylogenetic tree—and Hypothetical Taxonomic Unit (HTU)—typically associated to internal nodes in a phylogenetic analysis.

To facilitate more detailed validations, the concepts and relations are extended into corresponding disjoint (i.e. non-overlapping) sub-classes. The sub-class hierarchy is used to impose narrow limits on the domains and ranges of properties appropriate for each sub-class. Thus, a column in a protein sequence alignment is an ***AminoAcidResidueCharacter*** (subclass of ***MolecularCharacter: CategoricalCharacter: Character***). An individual ***TU*** for such a character has an ***AminoAcidResidueStateDatum*** (subclass of ***MolecularStateDatum: CategoricalStateDatum: CharacterStateDatum***) with a state-value drawn from the ***AminoAcidResidue*** domain (subclass of ***Molecular: Categorical: CharacterStateDomain***). Several generalized classes of characters are available, such as ***ContinuousCharacter****,* ***CategoricalCharacter*** and ***CompoundCharacter***.

##### (ii) Phylogenetic trees and networks

**Figure 4:**
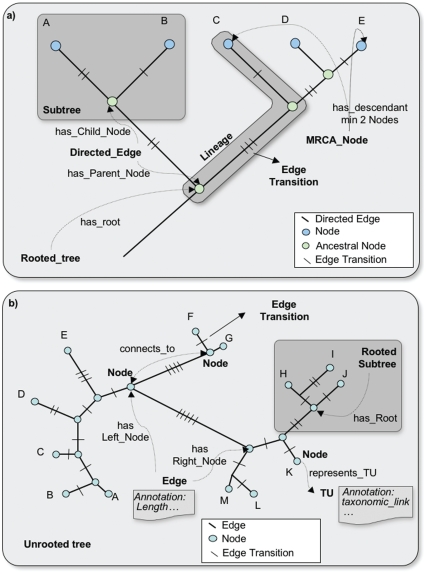
Annotation of rooted and unrooted evolutionary trees using CDAO concepts and relations. **a**) An example of a rooted tree showing how the concepts and relations defined in CDAO can be used to represent the topology of the tree and associated data. In particular, important evolutionary concepts, such as the Most Recent Common Ancestor (MRCA) can be specified. In the case of a rooted tree, the edges (or branches) of the tree are directed and the relations **has_parent_node** and **has_child_node** are used. **b**) The representation of an unrooted tree using CDAO. here, the direction of the edges is unknown and the relations **has_Left_node** and **has_Right_node** are used. Unrooted trees may contain subtrees for which the ancestor node is known, and in this case a rooted subtree can be specified using the **has_Root** relation.

In CDAO, phylogenetic trees and networks are made of ***Node***s and ***Edge***s (Fig. 4). A ***Node*** may be linked by the ***represents_TU*** property to a ***TU***, which represents a biological entity subject to evolutionary changes in its ***Character***s. An ***Edge*** represents the connection between two nodes in a tree or a network; it is described by the property ***has_Node***, which associates the edge to the nodes it connects, i.e.

*has_Node: Edge* → *Node*

An ***Edge*** has exactly two ***Nodes***, and this is encoded in the ontology through the mechanism of *superclass restriction*; using Protégé’s syntax, the class Edge is required to meet the requirement

has_Node exactly 2 Node

i.e. each Edge has exactly two properties linking it to a ***Node***.

***Tree*** has the subclasses ***Lineage***, ***RootedTree*** and ***UnrootedTree***. A ***RootedTree***, such as the one shown in Figure 2, has edges with a direction, called ***DirectedEdges***. The direction is described by replacing the generic ***has_Node*** property with the more specialized ***has_Child_Node*** and ***has_Parent_Node*** properties, which allow the edge to recognize the nodes it links as *parent* (i.e. ancestor node, closer to the root of the tree) or child. A ***RootedTree*** has also a root ***Node*** identified by the ***has_Root*** property.

CDAO describes different classes of trees, distinguished by their structures (e.g. ***RootedTree***, ***BifurcatingTree***, ***UnresolvedTree***) and by their properties (e.g. ***SpeciesTree***, ***ReconciledTree***).

##### (iii) Transitions, rules and models

The implementation of these concepts in CDAO is still incomplete, pending resolution of the ontological issue of how inferred or postulated evolutionary changes relate to phylogenetic trees (see Discussion for further comments on ontological questions to be resolved). Evolutionary transitions are currently treated as *annotations* of edges. Annotations (described by the generic class ***CDAOAnnotation***) can be associated to any concept, using the property

*has_Annotation: Thing* → *CDAOAnnotation*.

Several subclasses of ***CDAOAnnotation*** have been introduced, to describe annotations of different parts of a phylogenetic analysis. For instance, a ***ModelDescription*** (subclass of ***CDAOAnnotation***) can be used to describe the model of evolution used for the construction of the phylogenetic tree.

An ***EdgeTransition*** (subclass of ***EdgeAnnotation: CDAOAnnotation***) is linked to its ***Character***, i.e. the character affected by the transition, by the ***transition_of*** property:

*transition_of: EdgeTransition* → *Character*

The manner in which the ***EdgeTransition*** refers to its two character-states (the ones involved in the change, before and after) is subtle, due to the fact that an ***Edge*** may be an ***UndirectedEdge***. This prevents specifying a “before” and “after” state for the transition. In practice, the polarity or orientation of a transition relative to a tree is known even if the directionality of the tree’s edge (i.e. with respect to time) is not known. If a transition involving states 0 and 1 is postulated (or observed) on the edge connecting nodes A and B, then one intends that either the A side of the transition has state 1 and the B side has 0, or vice versa, but one does not intend an ambiguity. Therefore, we arbitrarily assign the orientation of a transition by the property ***has_Left_State*** and ***has_Right_State***, each of which refers to exactly one state from a ***CharacterStateDomain***. To complete the representation, one must specify which of the two nodes connected to the edge is considered to be the left node, and which one is the right node, using the ***has_Left_Node*** and ***has_Right_Node*** properties linking an ***EdgeTransition*** to its left and right ***Nodes***. To illustrate the transition concept, we can consider the ***CharacterStateData*** shown in Figure 2, where the *location* ***Character*** associated with TU “Dictyostelium_discodeum_AA051107.1” ***has_State*** “GO:0044425”. If we postulate that the ancestor of this TU ***has_State*** “GO:0016020”, then we can associate a transition with the ***Edge*** from “Dictyostelium_discodeum_AA051107.1” to its ancestor, where the ***EdgeTransition*** is ***transition_of*** location, ***has_Left_State*** “GO:0016020” and ***has_Right_State*** “GO:0044425”.

#### Representation of additional concepts in CDAO

The skeleton concept of a ***CoordinateSystem*** is provided to handle characters that are ordered in some way, e.g. residues in a nucleotide or protein sequence. In such cases, the coordinate system could refer to a sequence in an external database. Another example of a coordinate system would be the time (or order) coordinate for a developmental sequence.^67^ The CDAO coordinate system has a limited set of concepts for specifying locations, similar to the concepts used in NCBI’s data model.^55^

The ***CDAOAnnotation*** class can be used to describe annotations of different parts of a phylogenetic analysis. This class includes the previously mentioned ***EdgeAnnotation*** subclass—containing ***EdgeTransition***, along with a more generic edge length information, represented by the class ***EdgeLength—***and also several other subclasses: ***CharacterStateDataMatrixAnnotation***, ***TreeAnnotation***, ***TUAnnotation***, and ***ModelDescription***. Indeed, practitioners increasingly recommend including metadata in reports, as recommended in a recent call for a minimal reporting standard for a phylogenetic analysis.^53^ Certain kinds of metadata are considered particularly important, for example, the specific model of evolution used for the construction of the phylogenetic tree can be specified by a ***ModelDescription***. Another notable annotation is the association of OTUs with organismal sources specified in the terms of a taxonomy of species. In this case, CDAO provides a subclass of ***TUAnnotation***, called ***TaxonomicLink***, in order to refer to an external classification of biological species.

CDAO allows the representation of ***Lineage***s and most-recent-common-ancestors (***MRCANode***, subclass of ***Node***). Since OWL is limited to the representation of binary properties, an intermediate concept is required to represent the connection between a *collection* of nodes and the node that represents their most-recent common ancestor. This is realized by introducing a class ***SetOfNodes***. A concept from this class will be linked to the ***Node***s belonging to the set through the property ***has_Element***:

*has_Element: SetOfNodes* → *Node*

Given this intermediate concept, it is possible to associate a ***MRCANode*** to the ***SetOfNodes*** it is a most-recent-common ancestor of, using the property ***mrca_node_of***:

*mrca_node_of: MRCANode* → *SetOfNodes*

#### Integration of concepts from other ontologies

Evolutionary comparative methods are very general and can be applied to many different kinds of characters.^4^ In available artefacts, generality of data representation is achieved by a superficial treatment of the data as a matrix of arbitrary symbols, lacking the semantics needed to express rich biological knowledge of the characters and to ensure data consistency. For instance, in a probabilistic analysis of character evolution (e.g. using software such as^42^), the symbols “1” and “0” would be used to represent presence or absence (respectively) of a binary character, whether that character is a molecular character such as the presence or absence of an intron at gene site,^68^ or a non-molecular character such as the presence or absence of a soldier caste in an ant species.^69^

One way to support representation of the semantics of states would be to build in biological knowledge explicitly for each possible kind of character, including various types of molecular characters (e.g. sequence residues, enzyme activities, chemical entities) and higher-level characters (e.g. morphological and behavioral traits). However, this strategy is not realistic and would hinder the flexibility and useability of CDAO. Therefore, CDAO allows importing external ontologies, which can be used to provide biological knowledge relevant to reasoning about specific types of characters. The link can be made via the class ***CharacterStateDomain*** from which the states of a ***Character*** are drawn. In the current version of CDAO, this kind of link is exemplified by the treatment of amino acid residues. Although CDAO declares its own ***AminoAcidResidue*** subclass of ***CharacterStateDomain***, the specific amino acids are not declared within this CDAO class. Instead, this class is equated with the ***AminoAcid*** class imported from a pre-existing amino acid ontology (www.co-ode.org/ontologies/amino-acid). The imported ***AminoAcid*** class defines the 20 canonical amino acids (Alanine, Asparagine, …) as sub-classes, and has separate hierarchies for categories and qualities of amino acids, such as ***ChargedAminoAcid***. This illustrates the possibility to customize CDAO for a particular type of character by importing an ontology that supports specific forms of reasoning about that type of character.

### Evaluation

#### Coverage

The most recent version of CDAO includes 122 concepts, 67 object properties (i.e. properties that link instances of two concepts), and 10 data properties (i.e. properties that link instances of a concept to values of primitive data types such as integers). Compared to the concept glossary developed by the NESCent working group, CDAO includes 36 of the terms listed in the glossary; several other terms represent either synonyms or slight variations of terms present in the ontology. The remaining missing terms are mostly associated with the description of the actual evolutionary processes, while the current version of CDAO is focused on the informational concepts central to comparative analysis.

#### Representation

**Figure 5:**
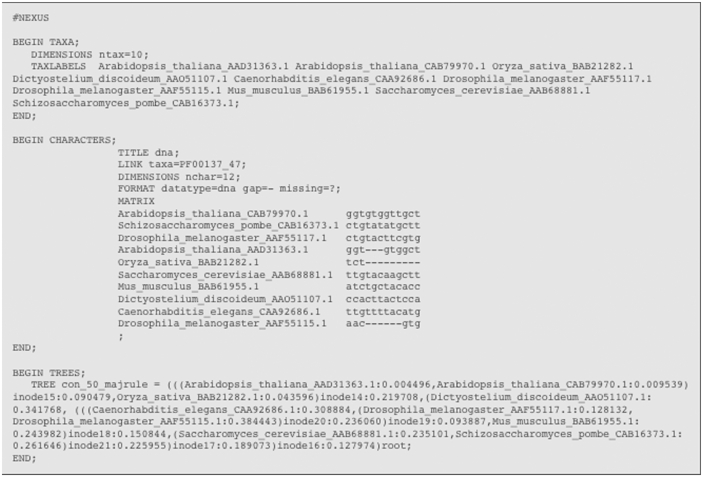
An example of instance data in the NEXUS format used commonly in phylogenetics.

A preliminary evaluation of the ability to represent domain-specific knowledge in CDAO has been carried out by translating comparative data from NEXUS^38^ files into instances of CDAO. The translation successfully maps data from the input files (described in Methods) to instances of CDAO classes and properties. This may be illustrated with the example of the NEXUS file of Figure 5, which represents the data shown in Figure 2. The CDAO encoding of a node (e.g. inode15), in RDF/XML notation, is


<**cdao:Node** rdf:ID=“inode15”>
          <**cdao:part_of** rdf:resource=“#Tree_con_50_majrule”/>
          <**cdao:belongs_to_Edge** rdf:resource=“#edge_inode15_inode14”/>
          <**cdao:belongs_to_Edge** rdf:resource=“#edge_A_thaliana_CAB79970_1_inode15”/>
          <**cdao:belongs_to_Edge** rdf:resource=“#edge_A_thaliana_AAD31363_1_inode15”/>
          <**cdao:belongs_to_Edge_as_Child** rdf:resource=“#edge_inode15_inode14”/>
          <**cdao:belongs_to_Edge_as_Parent** rdf:resource=“#edge_A_thaliana_CAB79970_1_inode15”/>
          <**cdao:belongs_to_Edge_as_Parent** rdf:resource=“#edge_A_thaliana_AAD31363_1_inode15”/>
          <**cdao:nca_node_of** rdf:resource=“#set_nca_44”/>
</**cdao:Node**>


The description includes the various properties applicable to this particular instance, e.g. those identifying the connection between the node and the incident edges, and the fact that this node is the most-recent-common ancestor for a group of nodes. The (directed) edge that links the A_thaliana_CAB79970.1 to inode15 is described as follows (note that the edge is annotated with length information):


<**cdao:Directed_Edge** rdf: ID=“edge_A_thaliana_CAB79970 _1_inode15”>
          <**cdao:part_of** rdf:resource=“#Tree”/>
          <**cdao:has_Parent_Node** rdf: resource=“#node_inode15”/>
          <**cdao:has_Child_Node** rdf resource=“#node_A_thaliana_CAB79970_1”/>
          <**cdao:has_Annotation** rdf:resource=“#edge_A_thaliana_CAB79970_1_inode15_length”/>
</**cdao:Directed_Edge**>
<**cdao:Edge_Length** rdf:ID=“edge_A_thaliana_CAB79970_1_inode15_length”>
          <**cdao:has_Value** rdf:datatype=“&xsd; float”>0.009539</**cdao:has_Value**>
</**cdao:Edge_Length**>


The encoding of the character state data matrix follows the same style. For example, the OTU C_elegans_ CAA92686.1 is described as


<**cdao:TU** rdf:ID=“C_elegans_CAA92686_1”>
          <**cdao:belongs_to_Character_State_Data_Matrix** rdf:resource=“#Matrix”/>
          <**cdao:represented_by_Node** rdf:resource=“#node_C_elegans_CAA92686_1”/>
          <**cdao:has_Nucleotide_Datum** rdf:resource=“#datum_C_elegans_CAA92686_1_char_0”/>
          <**cdao:has_Nucleotide_Datum** rdf:resource=“#datum_C_elegans_CAA92686_1_char_1”/>
          . . .
</**cdao:TU**>


The description includes a link to the node in the tree representing this TU and the links to the character state data associated with this TU (i.e. the cells of the row of the matrix). Each datum is associated with a TU and a character, and is linked to a representation of its state. For example, the datum for the previously described TU and the character number 6 is described as:


<**cdao:Nucleotide_State_Datum** rdf:ID=“datum_C_elegans_CAA92686_1_char_6”>
          <**cdao:belongs_to_Character** rdf:resource=“#char_6”/>
          <**cdao:belongs_to_TU** rdf:resource=“#C_elegans_CAA92686_1”/>
          <**cdao:has_Nucleotide_State** rdf:resource=“#A”/>
</**cdao:Nucleotide_State_Datum**>


#### Support for reasoning

Another way to evaluate CDAO is to consider its support for reasoning. We have carried out a preliminary evaluation of the ability to draw inferences from translated data (described above) using a reasoning engine for the OWL language.^37^,^47^ We employed two classes of queries to inspect the CDAO instances. The first set of queries has been developed within Protégé itself, using the FaCT++ reasoner plug-in.^46^ The Protégé querying interface allows the user to create new views of the ontology instances by defining new classes (using the existing classes and properties). In the previous example, a query to determine which TUs have at least one gap in their associated rows of the character state data matrix could be expressed as follows:

(*has_Datum* some (*has_State* value *gap*)) and *TU*

Intuitively, this reads as: *“collect all TUs that have a character state datum whose state has the value gap.”* In the example, this query will cause Protégé to list the instances that belong to this new class: D_melanogaster_AAF55115.1, A_thaliana_AAD31363.1, Oryza_sativa_BAB21282.1. Similarly, we can form a query to extract the ancestors of a node in the tree (e.g. D_melanogaster_AAF55115.1):

*has_Descendant* value node_D_melanogaster_AAF55115_1

where ***has_Descendant*** is a property obtained by performing a transitive closure of the ***has_Parent*** property.

The second set of queries has been developed using the SPARQL syntax and executed using the Pellet reasoner. Sample queries that have been tested are:

- Given a ***CharacterStateDatum***, determine the ***TU*** and the ***Character*** it belongs to
- Determine which ***TU***s and ***Character***s contain a gap
- Identify the subtree defined by two ***Node***s
- Given a ***TU***, retrieve its states for the various characters.

The reasoning tests uncovered several trouble spots where deficiencies in available reasoning engines required changes to the CDAO representation. While OWL 1.1 provides certain enhancements over previous versions of OWL—for example, we rely on the property chaining and transitivity axioms to implicitly define the ***has_Ancestor*** and ***has_Descendant*** properties—the Pellet reasoner does not always support them. As a consequence, changes were made to CDAO and the translator program to represent this information directly without using chaining. While most of the queries executed rapidly, certain queries (e.g. those that require matching data with the corresponding TU and Character) required a significant amount of time for large numbers of Characters and TUs.

## Discussion

The principles of evolutionary analysis follow from the assumption that descent-with-modification is the generating process for comparative biological data. Though powerful and generalizable, evolutionary analysis is difficult to apply in automatic systems. To make this approach more accessible to researchers, we have undertaken the development of a Comparative Data Analysis Ontology (CDAO). The initial implementation of CDAO, described here, covers key concepts required to perform evolutionary-based comparative analyses and has been evaluated for its capacity to support domain-specific representation and reasoning. CDAO is a SourceForge project and has a web home at www.evolutionaryontology.org/cdao. CDAO is implemented in OWL 1.1 to take advantage of the capabilities of description logics.

To understand the coverage and uses of CDAO, it is important to understand that it is not primarily an ontology of evolutionary processes or of evolutionary biology, but an ontology of evolutionary comparative analysis. The task-oriented nature of comparative analysis is apparent in concepts such as “OTU”— what defines something as an OTU is that it plays a particular role in an analysis. Thus, in CDAO, a ***TU*** (the generalization of OTU) is not restricted to refer to (to be about) any particular type of biological entity. Currently, CDAO provides terms for continuous characters, discrete characters, and several subclasses of discrete characters, including sequence characters. However, at present these classes remain very abstract, as CDAO does not import biological knowledge from other ontologies except the amino acid ontology mentioned above.

A challenge for the development of CDAO is to align its classes and relations with more fundamental concepts and relations, as consensus on these fundamentals begins to emerge from work in other areas of biology.^71^–^73^ As noted above, CDAO focuses on information artefacts rather than evolutionary processes. A phylogenetic tree clearly is not a biological entity or a process, but is more like a time-dependent model (a model in which time is one of the parameters) or a historical narrative. The relationship of such an artefact to a flesh-and-blood biological thing, e.g. the relationship of a terminal “cat” node on a phylogenetic tree to the concept of a cat, or cat species, is an issue that remains to be determined. The proper form of relationship to cross the boundary separating the universe of information artefacts from that of biological objects or processes might be something like “represents” or “is about”; clearly (by way of counter-example), the proper relation cannot be something like “has” or “part_of “. Even concepts that seem familiar in comparative analysis nonetheless pose difficult conceptual problems. When we see the state of a protein sequence character represented as “Ala” for “Alanine”, this does not mean precisely the free amino acid in solution, L-Alanine, because the proper constituent of a protein is the L-Alanyl moiety (i.e. CHEBI:32433 rather than CHEBI:16977).^30^ But even this is not quite right, because as a character state, the change from “Ala” to “Gly”, for instance, follows evolutionary rules, not strictly chemical rules; and even the non-change from “Ala” to “Ala” over time (e.g. lack of change over millions of years) is not a simple chemical preservation of a molecule. The “Ala” state is the state of an OTU that represents a population of gene-encoded proteins in some way that is difficult to grasp.

While it remains to be seen whether available upper-level ontologies are suitable for the complexity introduced by evolution, clearly they contain some useful concepts. For instance, the basic relation ontology OBO-REL^71^ refers to a formal relation of reproductive descendency, the relation that provides the continuity to an evolutionary lineage, i.e. a path in a tree. Likewise, the latest version of BioTop^73^ has a separate hierarchy including nucleotide residues as “informational” components of a sequences, as distinct from chemical compounds, a perspective that corresponds (in our understanding) to the way sequence data are treated in the context of evolutionary analysis. Nevertheless, a complete representation of comparative data analysis in terms of philosophically rigorous principles would seem difficult. Many important concepts in modern data analysis are not ontological in the sense of Smith,^74^ including concepts such as “posterior probability” and “annotation”. Thus, it may be appropriate to think of CDAO as an “application ontology” (or as a domain ontology that remains immature pending resolution of relevant philosophical issues).

A more practical challenge for the development of CDAO is to evaluate, revise and expand the ontology further, to ensure that it serves the purposes of comparative data analysis. As an ontology for comparative analysis, CDAO is designed to facilitate: integration of data from different resources; interoperation of different computational tools; creation of powerful software tools and methods based on evolutionary concepts; and interpretation of the results of comparative analysis by the non-expert.

Some of the representation challenges are foreseeable, such as fleshing out the ***CoordinateSystem*** concept to provide support for representation and reasoning about sequences (or other coordinate systems). Such an expansion is needed because, while CDAO allows the representation of sequence residues as character states, the columns of a character matrix do not have any inherent order, thus the sequence residues are not ordered in a sequence.

We have described here some initial tests for representation and reasoning, but a stronger test of CDAO will be performed in the context of projects with externally defined technical or scientific goals. The kinds of projects that are most demanding for data interoperability are integrative biology studies that attempt to integrate diverse data resources, while the kinds of projects that are most demanding for software interoperability are workflow systems that aim to provide access to diverse tools. Recently CDAO was made available for use during a Data Resource Interoperability Hackathon sponsored by the National Evolutionary Synthesis Center (March 9 to 13, 2009; http://evoinfo.nescent.org/Database_Interop_Hackathon). One group of participants used CDAO concepts to anchor metadata annotations in NeXML^49^ data files. Another group translated NeXML files into CDAO RDF/XML format using XSLT technology (see “Availability of Products” above), then loaded the results into a “triple store” (a collection of subject-predicate-object statements) which was interrogated using logical queries. We expect that the evaluation and further development of CDAO will take place in the context of such projects. The wider scientific community, particularly those researchers already involved in evolutionary-based analyses, is invited to participate in the further evaluation and development of CDAO.

## Conclusions

CDAO (Comparative Data Analysis Ontology) is a well annotated ontology, expressed in OWL and providing coverage of key concepts in evolutionary comparative analysis. These key concepts pertain to (i) phylogenetic trees of entities-to-be-compared; (ii) character-state data representing the compared attributes of entities; and (iii) evolutionary changes (transitions) in these characters. CDAO is designed to facilitate data interoperability and, indirectly, to facilitate the broader use of evolutionary methods.

## Figures and Tables

**Table 1:** Some related artefacts from the domain of evolutionary analysis.

Name	Type (language)	Coverage	Reference
NEXUS	File format (text)	Character data (various types), trees, assumptions, sets, notes	38
NeXmL	File format (XML Schema)	Character data (various types), trees, models, meta-data	www.nexml.org^49^
CHADO	DB schema (SQL)	Sequences, genotypes, phenotypes, phylogenies	www.gmod.org/wiki/index.php/Chado^56^
TreeBase	DB schema (SQL)	Character data (various types), trees, meta-info on analyses	www.treebase.org^75^
MAO	Ontology (OBO)	Multiple alignments of DNA, RNA and protein sequences	bips.u-strasbg.fr/LBGI/mAo/mao.html^57^
NCBI Taxonomy	DB schema (SQL)	Organismal classification using the Linnean system	www.ncbi.nlm.nih.gov/Taxonomy/
NCBI data model	Object model (ASN.1)	DNA, RNA and protein sequences, features, and alignments	www.ncbi.nlm.nih.gov/IEB/ToolBox/SDKDOCS/DATAMODL.HTML^55^
PATO	Ontology (OBO, OWL)	Phenotypic and trait ontology	www.bioontology.org/wiki/index.php/PATo
GO	Ontology (OBO, OWL)	Terms for molecular function, biological process, cellular location	www.geneontology.org^76^
SO	Ontology (OBO, OWL)	Sequence features and attributes, similarity, gene models	www.sequenceontology.org^29^
PRO	Ontology (OBO)	Protein entities, their structural parts, isoforms and modifications	pir.georgetown.edu/pro^77^
PO Protein ontology	Ontology (OWL)	Protein attributes other than sequence	proteinontology.info/78
RnaO	Ontology (OBO)	RNA sequence, structure, motifs, alignments	roc.bgsu.edu/79
